# A study on the physicochemical parameters for *Penicillium expansum* growth and patulin production: effect of temperature, pH, and water activity

**DOI:** 10.1002/fsn3.324

**Published:** 2015-12-16

**Authors:** Joanna Tannous, Ali Atoui, André El Khoury, Ziad Francis, Isabelle P. Oswald, Olivier Puel, Roger Lteif

**Affiliations:** ^1^ Unité de Technologie et Valorisation AlimentaireCentre d'Analyses et de RechercheUniversité Saint‐JosephCampus des Sciences et TechnologiesMar Roukos, Mkallès, P.O Box 11‐ 514, Riad El Solh1107 2050BeirutLebanon; ^2^ Research Centre in Food ToxicologyINRAUMR 1331 Toxalim180 Chemin de TournefeuilleF‐31027Toulouse CedexFrance; ^3^ Université de Toulouse IIIENVTINPUMR 1331ToxalimF‐31076ToulouseFrance; ^4^ Laboratory of Microorganisms and Food IrradiationLebanese Atomic Energy Commission‐CNRSP.O. Box 11‐8281, Riad El Solh1107 2260BeirutLebanon; ^5^ Department of BiologyFaculty of SciencesLaboratory of MicrobiologyLebanese UniversityHadath CampusBeirutLebanon

**Keywords:** Growth rate, patulin production, *Penicillium expansum*, pH, predictive mycology, temperature, water activity

## Abstract

*Penicillium expansum* is among the most ubiquitous fungi disseminated worldwide, that could threaten the fruit sector by secreting patulin, a toxic secondary metabolite. Nevertheless, we lack sufficient data regarding the growth and the toxigenesis conditions of this species. This work enables a clear differentiation between the favorable conditions to the *P. expansum* growth and those promising for patulin production. A mathematical model allowing the estimation of the *P. expansum* growth rate according to temperature, *a*_W_, and pH, was also developed. An optimal growth rate of 0.92 cm/day was predicted at 24°C with pH level of 5.1 and high *a*_W_ level of 0.99. The model's predictive capability was tested successfully on artificial contaminated apples. This model could be exploited by apple growers and the industrialists of fruit juices in order to predict the development of *P. expansum* during storage and apple processing.

## Introduction

Filamentous fungi are broadly dispersed throughout the environment and are responsible for the spoilage and poisoning of several food matrices. The most common and widespread mycotoxigenic fungi are mainly triggered by the genera: *Aspergillus*,* Fusarium,* and *Penicillium* (Sweeney and Dobson 1999; Binder et al. 2007). Within the latter genus, *Penicillium expansum* is one of the most studied species (Andersen et al. 2004). *Penicillium expansum* is a wound parasite fungus that invades fruits via injuries, caused by unfavorable weather conditions before harvest (hail, strong wind) or by rough handling, harvesting, and transport (Sanderson and Spotts 1995). This ubiquitous fungus commonly found on pome fruits causes a serious postharvest disease known as blue mold rot and produces significant amounts of patulin, giving rise to substantial fruit losses and serious public health issues (Moake et al. 2005). Patulin is known to have potent cytotoxic, genotoxic as well as immunotoxic effects even at relatively low exposure levels (Puel et al. 2010). Therefore, the European Union has fixed a maximum tolerated level of 50 μg/kg for fruit juices and derived products and 25 μg/kg for solid apple products. The maximum level allowed for apple products intended for infants and young children was set at 10 μg/kg (European C, 2003, 2006).

The understanding of *P. expansum* physiology under controlled experimental conditions may help forecast its behavior in natural conditions and predict its potential risks on the fruit sector and consumer health. In the last decades, predictive microbiology has emerged to be a useful tool in food industry used to predict the behavior of microorganisms through the development of several mathematical models capable of describing the responses of these pathogenic organisms to particular environmental conditions (Ross and McMeekin 1994; Fakruddin et al. 2011). Although it was more commonly used to control the bacterial growth (Gibson et al. 1988; Baranyi and Roberts 1994; Gaillard et al. 1998; Juneja et al. 2007), the situation has changed and this tool was lately employed in the modeling of fungal growth as well. The fungal proliferation and mycotoxin synthesis in foodstuffs are subject to multiple physicochemical parameters. The water activity (*a*_W_), and the temperature adopted during the storage period deemed as the most imperative ones (Holmquist et al. 1983; Dantigny et al. 2005; Bryden 2007). Likewise, other intrinsic factors, particularly the pH of the product, can largely affect the mold development (Rousk et al. 2009). The combination of these physicochemical parameters along with the usage of modeling techniques might be helpful to control the fungal growth and subsequently the biosynthesis of mycotoxins.

A growing number of studies are available in the literature dealing with the predictive modeling approach of fungi (Valık et al. 1999; Panagou et al. 2003; Parra and Magan 2004; Tassou et al. 2007; Garcia et al. 2011). For *P. expansum* in particular, few studies have been conducted to characterize the growth and the toxigenesis conditions of this species despite its large implication in foodstuff contamination. The growth rate of *P. expansum* has been studied as function of the storage temperature, the *a*_W_ and the oxygen levels (Lahlali et al. 2005; Marín et al. 2006; Baert et al. 2007a; Judet‐Correia et al. 2010).Moreover, its patulin production capacity has been independently assessed as a function of temperature, pH, and fruit varieties (Morales et al. 2008; Salomao et al. 2009). All these studies lack sufficient information about the simultaneous effects of such parameters on *P. expansum* growth and its patulin production capability. In this regard, it is worth mentioning that the most suitable conditions for the fungal growth may not be the optimal conditions for mycotoxin production, thus it is not possible to predict the latter from the kinetic growth data. Moreover, the interactive effects of different sets of abiotic factors cannot be predicted by such types of studies.

With these perspectives, this study was undertaken to firstly determine *in vitro* the individual effects of three major physicochemical parameters; the temperature, pH, and *a*_W_ on both the growth and patulin production by the blue‐rot ascomycetous fungus, *P. expansum*. These data were subsequently invested in the development of a mathematical model which enables accurate prediction of optimal and marginal conditions for *P. expansum* growth.

## Experimental

### Fungal isolate

This study was carried out on one strain of *P. expansum*, initially isolated from grapes in the Languedoc Roussillon region of France. The strain was previously characterized by DNA sequencing of the ITS gene region and deposited in ARS collection (USDA, Peoria, IL) as NRRL 35695. The strain was formerly confirmed as a patulin‐producer (Tannous et al. 2014).

### Experimental setup

#### Inoculum preparation

The investigated strain was subcultured on Potato Dextrose Agar (PDA) medium (Biolife, Milano, Italy) and incubated at 25°C to obtain a heavily sporulating culture. The conidial suspension was prepared by washing the surface of the fresh, mature (7‐day‐old colony) culture with 10 mL of sterile distilled water amended with Tween 80 (0.05%, v:v) and by gently rubbing with a sterile loop. The spores' concentration was reckoned by microscopy using a Neubauer counting chamber, and then adjusted to 10^5^ spores/μL.

#### Growth media and incubation conditions

All the assays were conducted on the synthetic Czapek Glucose agar medium in order to minimize other sources of variation that could be encountered on natural media and to identify clearly the effects of temperature, pH, and *a*_W_. This medium has already been proven to be a favorable substrate for *P. expansum* growth and patulin production (data not shown).

The overall assayed conditions were five temperatures, three pH levels, and four *a*_W_ values. Six separate replicate Petri plates were used for each temperature, *a*_W,_ and pH value, three of which were overlaid with sterilized cellophane disks to ensure a good separation between mycelium and agar. This will allow an accurate estimation of the mycelial mass and the amount of patulin produced on agar medium (Reeslev and Kjoller 1995; Tannous et al. 2014).

In all the experimental conditions, media were centrally inoculated with 10^6^ spores from the spore suspension. For temperature investigations, the synthetic Czapek glucose agar medium was prepared based on the formulation reported by Puel et al. (2005). The inoculated Petri plates were incubated at 30, 25, 16, 8, and 4°C in high precision (±0.1°C) for 2 weeks.

The synthetic Czapek glucose agar medium was also used for assessing the effect of *a*_W_ on the growth and patulin production by *P. expansum*. The unmodified medium (*a*_W_ 0.99) was adjusted to *a*_W_ levels of 0.95, 0.90, and 0.85 by adding increasing amounts of glycerol. Water activities were subsequently determined with the HygroLab 2 water activity indicator (Rotronic, Hauppauge, NY). Petri plates of the same *a*_W_ value were separately enclosed in polyethylene bags to prevent water loss. The inoculated Petri plates were incubated at 25°C for 2 weeks.

The pH surveys were also conducted on Czapek glucose agar incubated at a constant temperature of 25°C for only 7 days. The pH of the medium was adjusted to 2.5, 4, and 7 using two buffer solutions (Citric acid (0.5 mol/L): Potassium Hydrogen Phosphate (0.5 mol/L)) in the respective combinations 49 mL: 2 mL, 30.725 mL: 38.55 mL, and 8.825 mL: 82.35 mL, for a total volume of 250 mL of medium. These pH values were chosen as they cover the pH range found in different eating‐apple and cider apple varieties. The final pH of the medium was verified using a pH‐meter with special probe for alimentary articles by Hanna instruments (Tanneries, France).

### Growth and lag phase assessment

After inoculation, agar plates, harvested without cellophane disks, were checked on a daily basis to perceive if visible growth had started. As soon as a visible growth has begun, *P. expansum* growth was monitored by diameter measurements along two perpendicular directions, at regular time intervals. The lag phase (time required for growth) was evaluated and the radial growth rate (cm/day) was obtained from linear regression slopes of the temporal growth curves. Measurements were carried for an overall period of 14 days for the temperature and *a*_W_ surveys and 7 days only for the pH surveys.

Fungal growth was also evaluated with regard to biomass (mg dry weight). After the appropriate incubation period, the mycelia developed on the surface of agar plates topped with cellophane disks were scratched with a scalpel, collected, and dried at 80°C until a constant weight, corresponding to the dry biomass weight, was obtained.

### Patulin extraction and HPLC analysis

After the appropriate incubation period (7 days for the pH assays and 14 days for the temperature and *a*_W_ assays), the agar medium was scraped off the Petri dishes overlaid with sterile cellophane, cut into strips, mixed with 50 mL of ethyl acetate (Sigma‐Aldrich, Saint‐Quentin Fallavier, France) and macerated with agitation (250 rpm) at room temperature on an orbital shaker (Ningbo Hinotek Technology, Zhejiang, China). The contact time was 2 days. The organic phase was then filtered through Whatman Grade 413 filter paper (Merck, Darmstadt, Germany) and evaporated to dryness under liquid nitrogen. The dried residue was dissolved in 2 mL of methanol and then filtered through a 0.45 μm syringe filter (Navigator, Huayuan Tianjin, China) into a clean 2 mL vial. One hundred microliter aliquots of these extracts were injected onto the Waters Alliance HPLC system (Saint‐Quentin‐en‐Yvelines, France) for the quantitative determination of the patulin concentration. The patulin was detected with a Waters 2998 Photodiode Array Detector, using a 25 cm × 4.6 mm Supelco 5 μm Discovery C18 HPLC Column (Sigma‐Aldrich) at a flow rate of 1 mL/min. A gradient program was used with water (Eluent A) and acetonitrile HPLC grade (eluent B) and the following elution conditions: 0 min 5% B, 16 min 2% B, 20 min 60% B, 32 min 5% B. The presence of patulin was monitored at a 277 nm wavelength. A calibration curve was constructed with patulin standard (Sigma‐Aldrich) at concentrations ranging from 0.05 to 10 μg/mL. Accordingly, the patulin concentrations were determined and results were expressed in ppm. The LOD and LOQ of the method were calculated using the slope (S) of the calibration curve, obtained from linearity assessment, and the standard deviation of the response (SD). These values were determined as follows: LOD = 3.3 × SD/S, LOQ = 10 × SD/S.

### Model development

Growth rate experimental data were implemented in a home developed C++ language program that is able to interpolate between various points in different or multiple dimensions. Effects of the different parameters (temperature, pH, and *a*_W_) on the *P. expansum* growth rate were taken into account according to the experimental points already obtained. Thus, the effect of each of these parameters was considered on its own calculating the growth ratio factor effect obtained from the experimental data. The program proceeds as a simultaneous interpolator between the different data points and the effect ratios of each parameter on the growth rate. Therefore, the program allows us to estimate the growth rates (expressed in cm/day) for fixed temperature, *a*_W,_ and pH values depending on the variations defined by the input data. The model took into account the latency phase versus the temperature, which was modeled by a 4^th^ degree polynomial equation:

(1) $$\begin{array}{cc} \text{Latency}\text{(days)}= & 5.10^{-5}T^{4}-0.0039T^{3}+0.1126T^{2}\\ & -1.8349T+11.613 \end{array}$$

where *T* is the temperature parameter expressed in Degree Celsius (°C).

Likewise, the latency versus the *a*_W_ was taken into account to fit the following power equation:

(2) $$\text{Latency (days)}=0.9046a_{w}^{-12.98}$$

where *a*_w_ represents the water activity of the medium.

In the both latency phase fits, the correlation parameter *R* ^2^ was higher than 0.97 showing a good accuracy of the fitting procedure.

The *P. expansum* diameter growth (cm) versus time should theoretically follow a linear regression while considering the variation in each of the temperature, *a*_W_, and pH parameters. Therefore, a Pearson chi‐squared test was performed confirming that our hypothesis is true for over 99.9%. Thus, the slope and the intercept dependencies on each of the previously mentioned parameters were calculated according to the available experimental points.

Growth slope and intercept dependencies on temperature were fit into the following 4^th^ degree polynomial equations:

(3) $$\begin{array}{cc} \text{Slope(cm/day)}= & -3.10^{-5}T^{4}+0.0015T^{3}-0.0291T^{2}\\ & +0.2402T-0.368 \end{array}$$

(4) $$\begin{array}{cc} \text{Intercept (cm)}= & 10^{-5}T^{4}-0.0006T^{3}+0.006T^{2}\\ & +0.0536T-0.8269 \end{array}$$

The growth slope and intercept dependencies on *a*_W_ were fit into the following equations:

(5) $$\text{Slope (cm/day)}=5.2634a_{w}-4.46936$$

(6) $$\begin{array}{cc} \text{Intercept (cm)}= & 3.94927.10^{2}a_{w}^{3}-1.014943.10^{3}a_{w}^{2}\\ & +8.61994.10^{2}a_{w}-2.41545.10^{2} \end{array}$$

And the growth slope dependency on pH was fit into the following equation, considering the value for intercept as null:

(7) $$\text{Slope (cm/day)}=0.0578{\text{pH}}^{2}+0.584\text{pH}-0.5947$$

All slope and intercept fits were convergent to more than 99% with the experimental points.

In order to analyze the simultaneous effect of the different parameters, growth rate values were calculated for an *a*_W_ level of 0.99 and a pH of 4 (reference values) using the temperature's formula given by equations (3) and (4). Using these equations the growth rate (cm/day) can be calculated for different temperature values. Using the same method of proceeding we can use equations (5) and (6) to calculate the effect of the *a*_W_ on the growth rate and the equation (7) to analyze the effect of the pH. If two or more parameters are to be changed at the same time, the temperature effect is taken into account first, and then the obtained growth rate is further modified by the second parameter effect. The modification is a simple ratio factor that is applied to the growth rate following temperature change. Therefore, the effects of the *a*_W_ and the pH were implemented as a diameter ratio factor. Each factor was calculated by dividing the diameter obtained for a desired parameter value by that obtained for the experimental values that we considered as a reference.

Finally, a routine test of the different values combinations of pH, T, and *a*_W_ was carried out in order to retrieve the highest growth rate and its relative optimal parameters to obtain such a result.

### Validation of the predictive model in vivo

In order to assess the validity of the predictive model in natural conditions, three apple varieties (Golden Delicious, Granny Smith, and Royal Gala) with different initial pH values (Table 1) were used. As previously described by Sanzani et al. (2012), apples were surface‐sterilized using a 2% sodium hypochlorite solution and rinsed with water. Apples were then injured using a sterile toothpick to a depth of approximately 0.5 cm, and the wounded sites were inoculated with a 10 μL droplet of the *P. expansum* conidial suspension at a concentration of 10^5^ conidia/μL. The infected apples were then incubated for 2 weeks under three different temperatures (4°C, 25°C, and 30°C). The set of experimental conditions used to check the predictive capability of the model are given in Table 1. Duplicate analyses were performed on each set of conditions.

**Table 1:** Validation set of conditions of the predictive model for *Penicillium expansum* growth and patulin production

Apple variety	Temperature (°C)	Water activity	pH
Golden Delicious	4	0.98	3.5–3.6
	25	0.98	3.5–3.6
	30	0.98	3.5–3.6
Granny Smith	4	0.98	3.1–3.2
	25	0.98	3.1–3.2
	30	0.98	3.1–3.2
Royal Gala	4	0.99	4.1–4.2
	25	0.99	4.1–4.2
	30	0.99	4.1–4.2

The diameters of the rotten spots were measured daily and the experimental growth rates were estimated. To evaluate the performances of the developed model, the observed and predicted values were compared by plotting predicted growth rates against the experimental values.

### Statistical analysis

All values are stated as mean ± SEM unless otherwise indicated. For statistical analysis, the one‐way analysis of variance (ANOVA) was used (*P < 0.05; **P < 0.01; ***P < 0.001).

## Results and Discussion

### Studies on the growth and patulin production by *P. expansum* under different conditions

**Figure 1:**
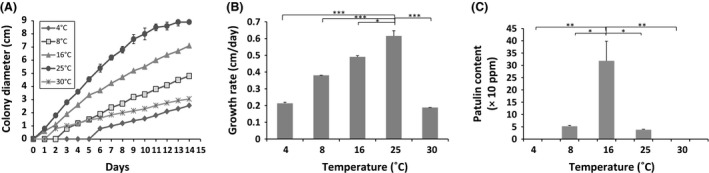
Growth curves (A), radial growth rates (cm/day) (B), and patulin production (ppm) of *Penicillium expansum* NRRL 35695 on Czapek glucose agar medium under different temperatures. Five different temperatures were tested (4°C, 8°C, 16°C, 25°C, and 30°C) with pH and *a*_W_ values fixed to 5.2 and 0.99, respectively. The results shown are the mean of three technical replicates for each condition. The standard errors of the mean (SEM) are represented by error bars: *P < 0.05; **P < 0.01; ***P < 0.001.

Colony diameters were measured on a daily basis and plotted against time. For all the tested conditions, the growth curves based on colony diameters were typical of a linear fungal growth after a short lag period ranged from 1 to 7 days (Baert et al. 2007a). However, it is worth mentioning that the fungal growth was in some cases limited by the Petri plates' dimension. In such cases, growth curves lose their linear appearance just after reaching the limiting diameter value (~7 cm) (Figs. 1A, 3A). Under each culture condition, the patulin content was quantified by HPLC and expressed in ppm.

#### Temperature effect

**Figure 2:**
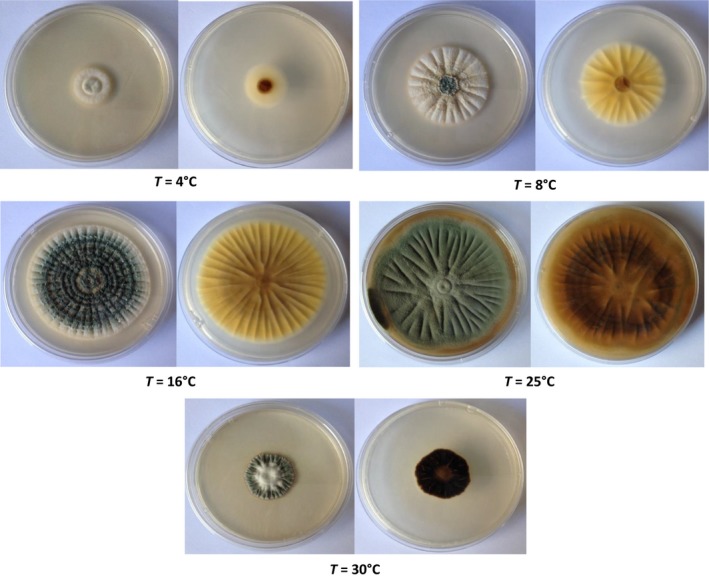
The colony appearance (surface and reverse) of *Penicillium expansum* isolate NRRL 35695 after 14 days of growth on Czapek glucose agar media under a wide range of temperatures.

Since in many cases, apples and other fruits are stored in refrigerators (at 4°C) or in plastic barrels outdoors where temperatures of 25–30°C are very common, the temperature analysis were performed within a 4 to 30°C range. The investigated strain of *P. expansum* was able to grow in the temperature range studied at unmodified *a*_W_ and pH (Fig. 1A). Interestingly, the strain displayed a different colonial morphology under the five tested temperatures. At 8°C and 16°C, green colony with white margins and yellow to cream reverse side was observed, whereas at 25°C, the fungus showed green conidia with dull‐brown color on reverse. An unusual morphology of the fungus was perceived at 30°C; the colonies grew vertically and stayed smaller than 3 cm, with serrated edges (Fig. 2). These morphological changes noticed following the incubation under various temperatures have been linked to stress response in other filamentous fungi (Verant et al. 2012).

The optimal temperature for the growth of this strain of *P. expansum* was around 25°C, at which the fungus exhibited the shortest lag phase and the most important colony growth (8.9 cm), at the end of the incubation period (Fig. 1A). This observation is in accordance with the literature data that describes also an optimum growth temperature for this species near 25°C (Pitt et al. 1991; Lahlali et al. 2005; Baert et al. 2007a; Pitt and Hocking 2009). Lag phases prior to growth increased when temperature varied from optimum to marginal conditions; a lag phase of 6 and 3 days was noticed at the lowest temperatures of 4°C and 8°C, respectively, besides a 2‐day‐latency period perceived at the highest studied temperature (30°C) (Fig. 1A). This result supports the prediction of Baert et al. (2007a) that cold storage does not prevent the fruit deterioration by *P. expansum*, but just delays it.

The colony growth rates were calculated as the slope of the linear segment of each growth curve. The growth rate of *P. expansum* as a function of temperature appears to have a bell‐shaped distribution with an optimum at 25°C and an experimentally determined value of 0.67 cm per day (Fig. 1B). The growth features of *P. expansum* were also evaluated in terms of fungal biomass development. The highest mycelia dry weight of 160 ± 15 mg per 20 mL of Czapek glucose medium was obtained at 25°C, followed by 16°C (130 ± 0 mg), 30°C (120 ± 10 mg) and 8°C (60 ± 10 mg). The lowest mycelia dry weight of 10 ± 0 mg was perceived at 4°C.

Patulin was identified by its retention time (9 min) and its UV spectra according to an authentical standard and quantified by measuring peak area according to the constructed standard curve of 0.919% coefficient of variation. The values of limit of detection (LOD) and limit of quantification (LOQ) for patulin were 0.04 μg/mL and 0.1 μg/mL, respectively. The patulin production by *P. expansum* exhibited also a marked temperature‐dependent variability. The histogram of patulin production versus temperature seen in Figure 1C has a characteristic bell shape. The highest patulin concentrations were attained at 16°C. However, a further increase in temperature to 25°C and 30°C caused a decrease in patulin production. These data matched a previous study of Paster et al. (1995) that compared patulin production on apples kept at various storage temperatures of 0, 3, 6, 17, and 25°C. In this study, the highest patulin concentration was found at 17°C. Our results are also in perfect agreement with those of Baert et al. (2007b) that showed a higher patulin production at low temperatures. However, they contradict many other studies that have reported a stimulation of the patulin production by this fungus by increasing the temperature (McCallum et al. 2002; Salomao et al. 2009). These results proved that temperature plays a role in patulin accumulation but not in a determinant way, other extrinsic and intrinsic factors appear to interact.

A comparison of the obtained bell‐shaped dependencies of the *P. expansum* growth rates and patulin levels as a function of temperature (Fig. 1B and C) revealed that the temperature ranges required to produce patulin were different and more restrictive than those for growth. Similar results have been reported for other fungal species. The *Fusarium* molds associated with the production of trichothecene (T‐2 and HT‐2 toxins) have been reported to grow prolifically at temperatures ranging between 25 and 30°C with a low production of mycotoxins. However, high levels of mycotoxins were produced at low temperatures (10 to 15°C), associated with a reduced fungal growth (Nazari et al. 2014). Similarly, the optimal temperature for Fumonisin B1 production was lower than the optimal temperature for the growth of *Fusarium verticillioides* and *Fusarium proliferatum* (Marin et al. 1999). Another example of a narrower range of temperatures for toxin production when compared with fungal growth is shown by the accumulation of ochratoxin in barley by *Penicillium verrucosum*. The growth of this species was conceivable at temperatures fluctuating between 0°C and 31°C, whereas the ochratoxin A production was only detected in the temperature range 12–24°C (Northolt et al. 1979).

#### Effect of water activity

**Figure 3:**
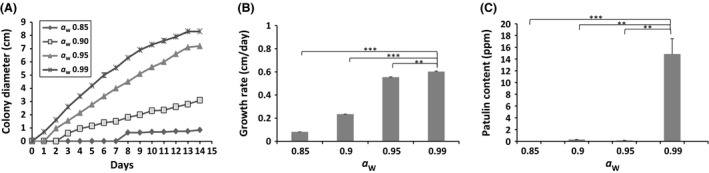
Growth curves (A), radial growth rates (cm/day) (B), and patulin production (ppm) of *Penicillium expansum* NRRL 35695 on Czapek glucose agar medium under modified water activity. Four different water activities were tested (0.85, 0.90, 0.95, and 0.99) with pH and temperature values fixed to 5.2°C and 25°C, respectively. The results shown are the mean of three technical replicates for each condition. The standard errors of the mean (SEM) are represented by error bars: *P < 0.05; **P < 0.01; ***P < 0.001.

The *a*_W_ of fresh fruits falls in the range 0.97–0.99. Though that patulin was also detected in dried fruits (Karaca and Nas 2006; Katerere et al. 2008) with *a*_W_ values less than 0.90, analyses were carried out over an *a*_W_ range 0.85–0.99. The Figure 3A shows the mean diameters of *P. expansum*, measured at different controlled *a*_W_, along culturing time. This species displayed an optimum growth at the highest *a*_W_ of 0.99, with the shortest lag phase and the most important colony growth (8.3 cm) after incubation period. For this highest value of *a*_W_, the fungus recorded the highest growth rate (0.6 cm per day). A drastic decrease in the *P. expansum* growth rate was observed by lowering the *a*_W_ from 0.99 to 0.85, using glycerol as humectant (Fig. 3B). The *P. expansum* isolate displays a different mycelial mass production in the Czapek glucose medium with modified *a*_W_. After 14 days, the highest production of fungal dry mass was obtained at the *a*_W_ of 0.95 (400 ± 50 mg dry weight per 20 mL of medium), followed by 0.99 (201 ± 10 mg) and 0.90 (105 ± 15 mg). A weak mycelium growth (1.1 ± 0 mg fungal dry weight) was reported at the minimal *a*_W_ tested. In literature, the minimal *a*_W_ for the germination of this species ranges between 0.83 (Mislivec and Tuite 1970) and 0.85 (Judet‐Correia et al. 2010), depending on the strain. As it can be observed, the mycelial dry weight estimated at the 0.95 *a*_W_ is approximately twice the value found at 0.99 *a*_W._ However, the fresh mycelial weight was significantly greater at the highest *a*_W_ value (data not shown).

The patulin production was also significantly affected by the water availability in the medium. No patulin was produced at an *a*_W_ of 0.85 throughout the incubation period. On the other hand, traces of patulin were detected after 14 days of culture, when the fungus was grown at the two *a*_W_ values of 0.90 and 0.95. A significant increase in the patulin production by *P. expansum* was perceived at the *a*_W_ of 0.99 (Fig. 3C). These findings on the impact of *a*_W_ on the patulin production by *P. expansum* are consistent with the two ancient studies reporting that the minimal *a*_W_ that allows patulin production by this fungus is of 0.95 (Lindroth et al. 1978; Patterson and Damoglou 1986).

As previously outlined for the temperature analysis, the *a*_W_ conditions that promote patulin production were also more restrictive than those allowing growth. Although there were no significant differences in terms of *P. expansum* growth at both water activities 0.95 and 0.99, the patulin production was significantly stimulated at 0.99, whereas only traces of patulin were detected at 0.95 (Fig. 3B and C).

#### Effect of pH

As several previous studies have reported a decrease in the pH of the medium during *P. expansum* growth, the pH assays were conducted on an overall incubation period of 7 days, in order to reduce pH fluctuations. This pH decrease is due to organic acids (gluconic acid) production, that lower the pH to values in which patulin is more stable (Baert et al. 2007b; Morales et al. 2008; Barad et al. 2013). In our study, the pH of the medium was recorded at the end of the experiment. The initial pH 7 slightly decreased to 6 along 7 days experiment; however, the two pH 2.5 and 4 were maintained constant at the initial value throughout the incubation period. The ability to change the ambient pH in order to generate a more suitable growing environment has been described for other fungal species and was shown to occur in either direction. Some necrotrophic species like *Alternaria alternata* (Eshel et al. 2002) or *Colletotrichum gloeosporioides* (Kramer‐Haimovich et al. 2006) can alkalize the host tissue by secreting ammonium, whereas other species can acidify the medium by secreting organic acids, like oxalic acid in the case of *Botrytis cinerea* (Manteau et al. 2003).

**Figure 4:**
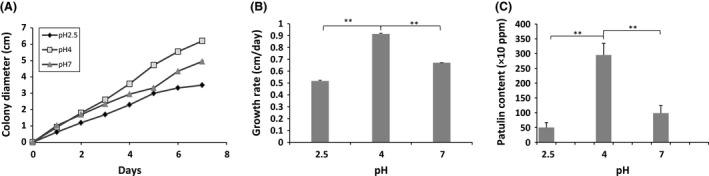
Growth curves (A), radial growth rates (cm/day) (B), and patulin production (ppm) of *Penicillium expansum* NRRL 35695 on Czapek glucose agar medium under modified pH. Three different pH values were tested (2.5, 4, and 7) with temperature and *a*_W_ values fixed to 25°C and 0.99, respectively. The results shown are the mean of three technical replicates for each condition. The standard errors of the mean (SEM) are represented by error bars: *P < 0.05; **P < 0.01; ***P < 0.001.

Under the different pH tested in our study, the lag phase periods were estimated to 1 day of culture from the linear regression curves for colony radius plotted versus time (Fig. 4A). It was also found that the growth rate of *P. expansum* as a function of pH is bell‐shaped with a maximum estimated value of 0.9 cm per day at pH 4 (Fig. 4B). Regarding, the mycelium dry weights, there were no significant differences between the three tested pH levels. Similarly, Morales et al. (2008) found that the *P. expansum* growth, estimated in terms of fungal biomass, was unaffected by the fruit juice initial pH.

The pH of the medium showed a significant effect on the ability of this fungus to produce patulin. The lowest patulin production was reported at pH 2.5, whereas the highest patulin level was detected at pH 4. The patulin‐producing capacity of *P. expansum* decreased when the pH of the medium increased from 4 to 7 (Fig. 4C). These results are comparable with those presented in previous studies. Damoglou and Campbell (1986) have previously reported that the pH range 2.8–3.2 resulted in less patulin accumulation by *P. expansum* compared to the pH range 3.4–3.8. While assessing the patulin accumulation in both apple and pear juices at different pH, Morales et al. (2008) have also observed an increase in patulin production by raising the pH from 2.5 to 3.5. The small amounts of patulin found at pH 2.5 are most probably due to a low production rather than to low stability of patulin. In the study of Drusch et al. (2007), the patulin stability was assessed over a wide pH range. Data from this study indicate that patulin is highly stable in the range pH 2.5–5.5. However, a greater decrease in the patulin concentration to 36% of the initial concentration was observed at neutral pH (Drusch et al. 2007).

### Mathematical modeling of *P. expansum* growth

The growth data modeled in this work comprised the latency phase and the growth curves of *P. expansum* strain NRRL 3565 at five temperatures, four *a*_W_, and three pH. Mycelial extension of colonies against time was almost invariable showing a straight line, after an initial lag period. The growth rates expressed in cm per day were calculated as the slope of the regression curves. The growth rates recorded under the different conditions (data presented above) were used as inputs to calculate the design parameters of equations (3), (4), (5), (6), (7).

**Figure 5:**
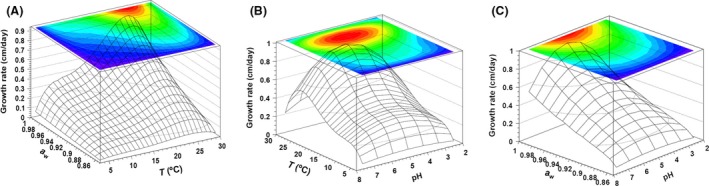
Three‐dimensional response surfaces showing the expected growth rates (cm/day) determined by the developed model as a function between temperature and *a*_W_ (A), temperature and pH (B), and pH and *a*_W_ (C). The graph A correspond to a fixed pH value of 4, the graph B to a fixed *a*_W_ value of 0.99 and the graph C to a fixed temperature of 25°C.

The presented calculation approach has undergone a first mathematical validation, confirming that the differences between the theoretical values predicted by the model and the data obtained under the conditions used to build the model are not significant. The Figure 5 shows the effects of temperature, *a*_W_ and pH on growth rate (expressed in cm/day) obtained using the approach described in this study. The surfaces generated by the model data summarizes all the growth rate values predicted under combined temperature and *a*_W_ (at a fixed pH 4), combined pH and temperature (at a fixed *a*_W_ of 0.99), and combined pH and *a*_W_ (at a fixed temperature of 25°C). The fixed values are those for which the effect of the other combination of factors on *P. expansum* growth is visualized the best. The model predicts that the optimal conditions for *P. expansum* growth were a temperature of 24°C, an *a*_W_ value of 0.99, and a pH value of 5.1. The predicted growth rate at optimal conditions was 0.92 cm/day. The minimal conditions for *P. expansum* growth as predicted by the model were a temperature of 3°C, an *a*_W_ value of 0.85, and a pH value of 2. Under these combined set of conditions, a slowdown of growth to almost zero level is predicted by the model.

**Figure 6:**
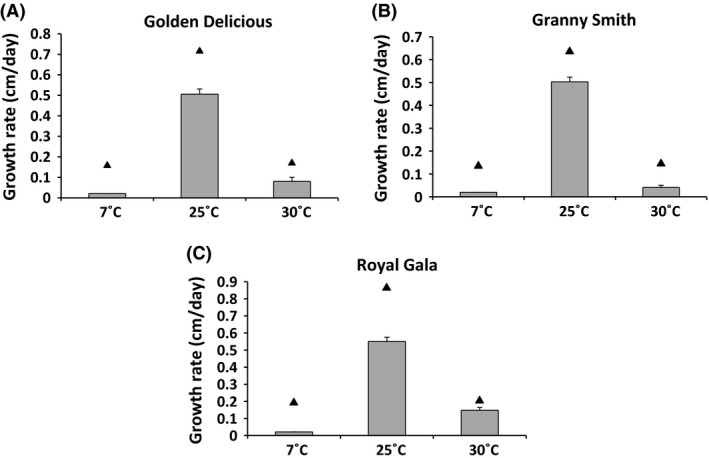
Comparison of the predicted and the observed *Penicillium expansum* growth responses on apples. The experimental growth rate values are shown in histograms (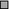), whereas the predicted values are represented by black triangles (▲). The experimental growth rate values are the average of two replicates for each condition with the standard deviations shown as error bars.

The experimental validation of the *P. expansum* growth model was carried out on apples. The objective was to test whether the performance of the predictive model may be low in a realistic situation or not. This validation led to acceptable results under most of the conditions. The growth of the fungus on apples was in general slower than predicted by the model (Fig. 6). However, using the Pearson product‐moment correlation coefficient, a value of 0.96 was found between the experimental and predicted growth rate values. The difference between the predicted and observed growth rates on apple is most probably due to the apple itself, which might be a stress factor for the fungus. Such stress factors include the intact tissue structure of the apple, which must be degraded in order to enable mold development to occur and which causes a reduced O_2_ availability within the fruit. A similar result was observed in a previous study investigating the effect of temperature on *P. expansum* growth in both Apple Puree Agar Medium (APAM) and apples (Baert et al. 2007a). Our results obtained from *in vivo* experiments indicate that the employment of the developed modeling approach, to assess the combined effect of temperature, *a*_W_ and pH on the growth responses of *P. expansum* could be satisfactory. However, the risk in using the described model in real situations may lie in the difference in the initial inoculum size and the unrealistic constant conditions of temperature and moisture content.

A review of the literature reveals that certain mathematical models were developed to describe and predict the *P. expansum* growth under different environmental conditions. The combined effects of temperature and *a*_W_ on the growth rate of *P. expansum* were previously studied and modeled by Lahlali et al. (2005) on PDA medium. In their study, the data obtained with both sorbitol and glycerol as humectant were modeled by means of the quadratic polynomial model. In agreement with our findings, it was shown that *P. expansum* grows best at temperatures ranging from 15 to 25°C and at an *a*_w_ ranging from 0.960 to 0.980. The growth rate and the lag time for six *P. expansum* strains were modeled as a function of temperature by Baert et al. (2007a) on APAM. In accordance with our results, the optimal temperature for growth varied between 24°C and 27°C depending on the strain. A similar modeling study was later conducted on both malt extract agar (with a pH value of 4.2 and an *a*_W_ of 0.997) and on simulating yogurt medium (Gougouli and Koutsoumanis 2010), where a Cardinal Model with Inflection (CMI) was used. The optimal temperature for *P. expansum* growth was determined as 22.08°C, which was close to that predicted by our model. Moreover, the predicted growth rate (0.221 mm/h, the equivalent of 0.55 cm/day), was lower than that expected in our study. Another predictive study was conducted by Judet‐Correia et al. (2010) using the Cardinal Model with Inflection. The objective of the latter was to develop and validate a model for predicting the combined effect of temperature and *a*_W_ on the radial growth rate of *P. expansum* on PDA medium. The optimal conditions estimated by this study on Potato Dextrose were 23.9°C for temperature and 0.981 for *a*_W_. These estimated values are close to those predicted in the present work. However, the optimal growth rate expected was remarkably lower. This difficulty in comparing the growth rates is obviously due to the fact that the isolates in the study of Judet‐Correia et al. (2010) were grown on PDA, whose composition differs from that of Czapek glucose agar.

The importance of this study resides in the fact that it takes into account the three key growth factors (Temperature, *a*_W_ and pH) unlike the previously conducted studies that did not consider more than two exogenous factors. It is also worth mentioning that the effect of the latter factor on *P. expansum* growth has never been modeled before. In addition to its growth modeling approach, this study considers the distinction between the favorable conditions for growth and those for toxigenesis of *P. expansum*.

It remains to note that this predictive model is built up based on the data on one *P. expansum* strain (NRRL 35695). As previously reported by McCallum et al. (2002), *P. expansum* isolates exhibit different growth rates. In this regard, it will be interesting to evaluate the ability of this model to extrapolate to other strains within the same species. Ultimately, the extent to which the model can be applied to other inoculum sizes, other growth media and fluctuating temperatures is to be determined in future validation studies for extrapolation.

## Conclusion

The findings in this study provide a considerable insight and a very interesting and informative comparison of the growth rate and patulin production of *P*. *expansum* regarding three eco‐physiological factors mainly involved in the proliferation of pathogenic fungi.

In the present work, a predictive model was also developed as a tool to be used for the interpretation of *P. expansum* growth rate data. Within the experimental limits of temperature, pH and *a*_W_, this model was able to predict the colony radial growth rates (cm/day) along a wide combination of culture conditions. Furthermore, the validation showed that the model can predict the growth of *P. expansum* under natural conditions on apples, with an acceptable accuracy. To conclude, the developed mathematical model for predicting the *P. expansum* growth on a laboratory scale can be used as a tool to assess the risk of *P. expansum* in fruit juices industry by predicting conditions over which the *P. expansum* growth in food matrices might be a problem.

## Conflict of Interest

None declared.

## References

[fsn3324-bib-0001] Andersen, B. , J. Smedsgaard , and J. C. Frisvad . 2004 *Penicillium expansum*: consistent production of patulin, chaetoglobosins, and other secondary metabolites in culture and their natural occurrence in fruit products. J. Agric. Food Chem. 52:2421–2428. 15080656 10.1021/jf035406k

[fsn3324-bib-0002] Baert, K. , A. Valero , B. De Meulenaer , S. Samapundo , M. M. Ahmed , L. Bo , et al. 2007a Modeling the effect of temperature on the growth rate and lag phase of *Penicillium expansum* in apples. Int. J. Food Microbiol. 118:139–150. 17698233 10.1016/j.ijfoodmicro.2007.07.006

[fsn3324-bib-0003] Baert, K. , F. Devlieghere , H. Flyps , M. Oosterlinck , M. M. Ahmed , A. Rajković , et al. 2007b Influence of storage conditions of apples on growth and patulin production by *Penicillium expansum* . Int. J. Food Microbiol. 119:170–181. 17900726 10.1016/j.ijfoodmicro.2007.07.061

[fsn3324-bib-0004] Barad, S. , S. B. Horowitz , I. Kobiler , A. Sherman , and D. Prusky . 2013 Accumulation of the mycotoxin patulin in the presence of gluconic acid contributes to pathogenicity of *Penicillium expansum* . Mol. Plant Microbe Interact. 27:66–77. 24024763 10.1094/MPMI-05-13-0138-R

[fsn3324-bib-0005] Baranyi, J. , and T. A. Roberts . 1994 A dynamic approach to predicting bacterial growth in food. Int. J. Food Microbiol. 23:277–294. 7873331 10.1016/0168-1605(94)90157-0

[fsn3324-bib-0006] Binder, E. M. , L. M. Tan , L. J. Chin , J. Handl , and J. Richard . 2007 Worldwide occurrence of mycotoxins in commodities, feeds and feed ingredients. Anim. Feed Sci. Technol. 137:265–282.

[fsn3324-bib-0007] Bryden, W. L. 2007 Mycotoxins in the food chain: human health implications. Asia Pac. J. Clin. Nutr. 16:95–101. 17392084

[fsn3324-bib-0008] Damoglou, A. P. , and D. S. Campbell . 1986 The effect of pH on the production of patulin in apple juice. Lett. Appl. Microbiol. 2:9–11.

[fsn3324-bib-0009] Dantigny, P. , A. Guilmart , and M. Bensoussan . 2005 Basis of predictive mycology. Int. J. Food Microbiol. 100:187–196. 15854704 10.1016/j.ijfoodmicro.2004.10.013

[fsn3324-bib-0010] Drusch, S. , S. Kopka , and J. Kaeding . 2007 Stability of patulin in a juice‐like aqueous model system in the presence of ascorbic acid. Food Chem. 100:192–197.

[fsn3324-bib-0011] Eshel, D. , I. Miyara , T. Ailing , A. Dinoor , and D. Prusky . 2002 pH regulates endoglucanase expression and virulence of *Alternaria alternata* in persimmon fruit. Mol. Plant Microbe Interact. 15:774–779. 12182334 10.1094/MPMI.2002.15.8.774

[fsn3324-bib-0012] European C . 2003 Commission regulation (EC) No 1425/2003 of 11 August 2003 amending regulation (EC) No 466/2001 as regards patulin. Off. J. Eur. Union L 203:1–3.

[fsn3324-bib-0013] European C . 2006 Commission Regulation (EC) No 1881/2006 of 19 December 2006 setting maximum levels for certain contaminants in foodstuffs. Off. J. Eur. Union L 364:5–24.

[fsn3324-bib-0014] Fakruddin, M. , R. M. Mazumdar , and K. S. B. Mannan . 2011 Predictive microbiology: modeling microbial responses in food. Ceylon J. Sci. Biol. Sci. 40:121–131.

[fsn3324-bib-0015] Gaillard, S. , I. Leguérinel , and P. Mafart . 1998 Model for combined effects of temperature, pH and water activity on thermal inactivation of *Bacillus cereus* spores. J. Food Sci. 63:887–889.

[fsn3324-bib-0016] Garcia, D. , A. J. Ramos , V. Sanchis , and S. Marín . 2011 Modelling the effect of temperature and water activity in the growth boundaries of *Aspergillus ochraceus* and *Aspergillus parasiticus* . Food Microbiol. 28:406–417. 21356445 10.1016/j.fm.2010.10.004

[fsn3324-bib-0017] Gibson, A. M. , N. Bratchell , and T. A. Roberts . 1988 Predicting microbial growth: growth responses of *Salmonellae* in a laboratory medium as affected by pH, sodium chloride and storage temperature. Int. J. Food Microbiol. 6:155–178. 3275296 10.1016/0168-1605(88)90051-7

[fsn3324-bib-0018] Gougouli, M. , and K. P. Koutsoumanis . 2010 Modelling growth of *Penicillium expansum* and *Aspergillus niger* at constant and fluctuating temperature conditions. Int. J. Food Microbiol. 140:254–262. 20413170 10.1016/j.ijfoodmicro.2010.03.021

[fsn3324-bib-0019] Holmquist, G. U. , H. W. Walker , and H. M. Stahr . 1983 Influence of temperature, pH, water activity and antifungal agents on growth of *Aspergillus flavus* and *A. parasiticus* . J. Food Sci. 48:778–782.

[fsn3324-bib-0020] Judet‐Correia, D. , S. Bollaert , A. Duquenne , C. Charpentier , M. Bensoussan , and P. Dantigny . 2010 Validation of a predictive model for the growth of *Botrytis cinerea* and *Penicillium expansum* on grape berries. Int. J. Food Microbiol. 142:106–113. 20619474 10.1016/j.ijfoodmicro.2010.06.009

[fsn3324-bib-0021] Juneja, V. K. , M. Valenzuela Melendres , L. Huang , V. Gumudavelli , J. Subbiah , and H. Thippareddi . 2007 Modeling the effect of temperature on growth of *Salmonella* in chicken. Food Microbiol. 24:328–335. 17189758 10.1016/j.fm.2006.08.004

[fsn3324-bib-0022] Karaca, H. , and S. Nas . 2006 Aflatoxins, patulin and ergosterol contents of dried figs in Turkey. Food Addit. Contam. 23:502–508. 16644598 10.1080/02652030600550739

[fsn3324-bib-0023] Katerere, D. R. , S. Stockenström , and G. S. Shephard . 2008 HPLC‐DAD method for the determination of patulin in dried apple rings. Food Control 19:389–392.

[fsn3324-bib-0024] Kramer‐Haimovich, H. , E. Servi , T. Katan , J. Rollins , Y. Okon , and D. Prusky . 2006 Effect of ammonia production by *Colletotrichum gloeosporioides* on pelB activation, pectate lyase secretion, and fruit pathogenicity. Appl. Environ. Microbiol. 72:1034–1039. 16461646 10.1128/AEM.72.2.1034-1039.2006 PMC1392887

[fsn3324-bib-0025] Lahlali, R. , M. N. Serrhini , and M. H. Jijakli . 2005 Studying and modelling the combined effect of temperature and water activity on the growth rate of *P. expansum* . Int. J. Food Microbiol. 103:315–322. 15885834 10.1016/j.ijfoodmicro.2005.02.002

[fsn3324-bib-0026] Lindroth, S. , A. Niskanen , and O. Pensala . 1978 Patulin production during storage of blackcurrant, blueberry and strawberry jams inoculated with *Penicillium expansum* mould. J. Food Sci. 43:1427–1429.

[fsn3324-bib-0027] Manteau, S. , S. Abouna , B. Lambert , and L. Legendre . 2003 Differential regulation by ambient pH of putative virulence factor secretion by the phytopathogenic fungus *Botrytis cinerea* . FEMS Microbiol. Ecol. 43:359–366. 19719667 10.1111/j.1574-6941.2003.tb01076.x

[fsn3324-bib-0028] Marin, S. , N. Magan , J. Serra , A. J. Ramos , R. Canela , and V. Sanchis . 1999 Fumonisin B1 production and growth of *Fusarium moniliforme* and *Fusarium proliferatum* on maize, wheat, and barley grain. J. Food Sci. 64:921–924.

[fsn3324-bib-0029] Marín, S. , H. Morales , A. J. Ramos , and V. Sanchis . 2006 Evaluation of growth quantification methods for modelling the growth of *Penicillium expansum* in an apple‐based medium. J. Sci. Food Agric. 86:1468–1474.

[fsn3324-bib-0030] McCallum, J. L. , R. Tsao , and T. Zhou . 2002 Factors affecting patulin production by *Penicillium expansum* . J. Food Prot. 65:1937–1942. 12495013 10.4315/0362-028x-65.12.1937

[fsn3324-bib-0031] Mislivec, P. B. , and J. Tuite . 1970 Temperature and relative humidity requirements of species of *Penicillium* isolated from yellow dent corn kernels. Mycologia 62:75–88. 4314984

[fsn3324-bib-0032] Moake, M. M. , O. I. Padilla‐Zakour , and R. W. Worobo . 2005 Comprehensive review of patulin control methods in foods. Compr. Rev. Food Sci. Food Saf. 4:8–21. 10.1111/j.1541-4337.2005.tb00068.x 33430570

[fsn3324-bib-0033] Morales, H. , G. Barros , S. Marín , S. Chulze , A. J. Ramos , and V. Sanchis . 2008 Effects of apple and pear varieties and pH on patulin accumulation by *Penicillium expansum* . J. Sci. Food Agric. 88:2738–2743.

[fsn3324-bib-0034] Nazari, L. , E. Pattori , V. Terzi , C. Morcia , and V. Rossi . 2014 Influence of temperature on infection, growth, and mycotoxin production by *Fusarium langsethiae* and *F. sporotrichioides* in durum wheat. Food Microbiol. 39:19–26. 24387848 10.1016/j.fm.2013.10.009

[fsn3324-bib-0035] Northolt, M. D. , H. P. Van Egmond , and W. E. Paulsch . 1979 Ochratoxin A production by some fungal species in relation to water activity and temperature. J. Food Prot. 42:485–490. 10.4315/0362-028X-42.6.485 30812257

[fsn3324-bib-0036] Panagou, E. Z. , P. N. Skandamis , and G.‐J. Nychas . 2003 Modelling the combined effect of temperature, pH and *a* _W_ on the growth rate of Monascus ruber, a heat‐resistant fungus isolated from green table olives. J. Appl. Microbiol. 94:146–156. 12492935 10.1046/j.1365-2672.2003.01818.x

[fsn3324-bib-0037] Parra, R. , and N. Magan . 2004 Modelling the effect of temperature and water activity on growth of *Aspergillus niger* strains and applications for food spoilage moulds. J. Appl. Microbiol. 97:429–438. 15239711 10.1111/j.1365-2672.2004.02320.x

[fsn3324-bib-0038] Paster, N. , D. Huppert , and R. Barkai‐Golan . 1995 Production of patulin by different strains of *Penicillium expansum* in pear and apple cultivars stored at different temperatures and modified atmospheres. Food Addit. Contam. 12:51–58. 7758631 10.1080/02652039509374278

[fsn3324-bib-0039] Patterson, M. , and A. P. Damoglou . 1986 The effect of water activity and pH on the production of mycotoxins by fungi growing on a bread analogue. Lett. Appl. Microbiol. 3:123–125.

[fsn3324-bib-0040] Pitt, J. I. , and A. A. D. Hocking . 2009 Fungi and food spoilage, 3rd edition Springer, United States.

[fsn3324-bib-0041] Pitt, J. I. , R. A. Spotts , R. J. Holmes , and R. H. Cruickshank . 1991 *Penicillium solitum* revived, and its role as a pathogen of pomaceous fruit. Phytopathology 81:1108–1112.

[fsn3324-bib-0042] Puel, O. , S. Tadrist , P. Galtier , I. P. Oswald , and M. Delaforge . 2005 *Byssochlamys nivea* as a source of mycophenolic acid. Appl. Environ. Microbiol. 71:550–553. 15640234 10.1128/AEM.71.1.550-553.2005 PMC544229

[fsn3324-bib-0043] Puel, O. , P. Galtier , and I. P. Oswald . 2010 Biosynthesis and toxicological effects of patulin. Toxins 2:613–631. 22069602 10.3390/toxins2040613 PMC3153204

[fsn3324-bib-0044] Reeslev, M. , and A. Kjoller . 1995 Comparison of biomass dry weights and radial growth rates of fungal colonies on media solidified with different gelling compounds. Appl. Environ. Microbiol. 61:4236–4239. 16535179 10.1128/aem.61.12.4236-4239.1995 PMC1388644

[fsn3324-bib-0045] Ross, T. , and T. A. McMeekin . 1994 Predictive microbiology. Int. J. Food Microbiol. 23:241–264. 7873329 10.1016/0168-1605(94)90155-4

[fsn3324-bib-0046] Rousk, J. , P. C. Brookes , and E. Bååth . 2009 Contrasting soil pH effects on fungal and bacterial growth suggest functional redundancy in carbon mineralization. Appl. Environ. Microbiol. 75:1589–1596. 19151179 10.1128/AEM.02775-08 PMC2655475

[fsn3324-bib-0047] Salomao, B. , G. M. F. Aragão , J. J. Churey , O. I. Padilla‐Zakour , and R. W. Worobo . 2009 Influence of storage temperature and apple variety on patulin production by *Penicillium expansum* . J. Food Prot. 72:1030–1036. 19517731 10.4315/0362-028x-72.5.1030

[fsn3324-bib-0048] Sanderson, P. G. , and R. A. Spotts . 1995 Postharvest decay of winter pear and apple fruit caused by species of *Penicillium* . Phytopathology 85:103–110.

[fsn3324-bib-0049] Sanzani, S. M. , M. Reverberi , M. Punelli , A. Ippolito , and C. Fanelli . 2012 Study on the role of patulin on pathogenicity and virulence of *Penicillium expansum* . Int. J. Food Microbiol. 153:323–331. 22189024 10.1016/j.ijfoodmicro.2011.11.021

[fsn3324-bib-0050] Sweeney, M. J. , and A. D. W. Dobson . 1999 Molecular biology of mycotoxin biosynthesis. FEMS Microbiol. Lett. 175:149–163. 10386364 10.1111/j.1574-6968.1999.tb13614.x

[fsn3324-bib-0051] Tannous, J. , R. El Khoury , S. P. Snini , Y. Lippi , A. El Khoury , A. Atoui , et al. 2014 Sequencing, physical organization and kinetic expression of the patulin biosynthetic gene cluster from *Penicillium expansum* . Int. J. Food Microbiol. 189:51–60. 25120234 10.1016/j.ijfoodmicro.2014.07.028

[fsn3324-bib-0052] Tassou, C. C. , E. Z. Panagou , P. Natskoulis , and N. Magan . 2007 Modelling the effect of temperature and water activity on the growth of two ochratoxigenic strains of *Aspergillus carbonarius* from Greek wine grapes. J. Appl. Microbiol. 103:2267–2276. 18045410 10.1111/j.1365-2672.2007.03480.x

[fsn3324-bib-0053] Valık, L. , J. Baranyi , and F. Görner . 1999 Predicting fungal growth: the effect of water activity on *Penicillium roqueforti* . Int. J. Food Microbiol. 47:141–146. 10357282 10.1016/s0168-1605(98)00201-3

[fsn3324-bib-0054] Verant, M. L. , J. G. Boyles , W. Jr Waldrep , G. Wibbelt , and D. S. Blehert . 2012 Temperature‐dependent growth of *Geomyces destructans,* the fungus that causes bat white‐nose syndrome. PLoS One 7:e46280. 23029462 10.1371/journal.pone.0046280 PMC3460873

